# 830 nm photobiomodulation therapy promotes engraftment of human umbilical cord blood-derived hematopoietic stem cells

**DOI:** 10.1038/s41598-020-76760-5

**Published:** 2020-11-12

**Authors:** Jingke Yang, Li Wang, Mei X. Wu

**Affiliations:** Department of Dermatology, Wellman Center for Photomedicine, Massachusetts General Hospital, Harvard Medical School, Boston, MA 02114 USA

**Keywords:** Biological techniques, Stem cells

## Abstract

Human umbilical cord blood (hUCB)-derived hematopoietic stem cells (HSCs) are an important source for HSCs in allogeneic HSC transplantation, but a limited number and a low efficacy of engraftment greatly restrict their clinical use. Here, we report the ability of photobiomodulation therapy (PBMT) to significantly enhance the engraftment efficacy of hUCB HSCs and progenitor cells (HSPCs). hUCB CD34^+^ cells were illuminated at a fluence of 2 J/cm^2^ with a near-infrared light (830 nm) transmitted by an array of light-emitting diodes (LED) prior to infusion of NOD/SCID-IL2Rγ^−/−^ mice. The pre-treatment resulted in a threefold higher of the mean percentage of human CD45^+^ cells in the periphery of the mice compared to sham-treated CD34^+^ cells. The enhanced engraftment may result from a PBMT-mediated increase of intracellular reactive oxygen species (ROS) levels and Src protein phosphorylation in CD34^+^ cells. The two events were causally related as suggested by the finding that elevation of ROS by hydrogen peroxide increased Src phosphorylation, while ROS reduction by N-acetyl cysteine partially reversed the phosphorylation. The investigation demonstrates that PBMT can promote engraftment of hUCB HPSCs, at least in part, via ROS-mediated Src signaling pathway. PBMT can be potentially a safe, convenient, and cost-effective modality to improve hematological reconstitution in patients.

## Introduction

Human umbilical cord blood (hUCB) contains plenty of hematopoietic stem cells (HSCs) in comparison with peripheral blood. Moreover, its easy accessibility through banking and low risk for graft-versus-host disease after transplantation make hUCB an important source for HSCs in allogeneic HSC transplantation^1^. However, a limited number of hUCB hematopoietic stem/progenitor cells (HSPCs) per donor and/or poor homing of HSPCs often lead to a failure of single-unit UCB transplantation and complications due to delayed engraftment and hematologic reconstitution^1,2^.

Several strategies have been adopted and explored to facilitate HPSC engraftment. Among them, double-unit UCB transplantation is a common one in the clinic, which is frequently accompanied with a high risk for early post-transplant complications^3^. Another potential strategy is to expand hUCB HSPCs ex vivo before transplantation by culturing HSPCs in the presence of various cytokines, stromal cells, small molecular agents, or transient gene modulation of HSC regulatory factors. Although some approaches in early clinical trials have increased the number of CD34^+^ cells resulting in a faster neutrophil recovery, such as Notch-based methods, whether these approaches could provide reliable engraftment remains unclear in clinics^4^. In recent years, enhancement of HSPCs homing to bone marrow presents new strategies to improve engraftment. Some molecules have been found to modulate homing via promoting migration and adhesion of HPSCs, such as chemokine CXCL12 (also known as stromal-derived factor 1)^5^. The chemokines-mediated promotion of HPSC homing is still in pre-clinical stages despite decade investigation, the safety and clinical value of which remain to be defined. Moreover, the cost would be a concern for all these strategies. Apparently, there is a need to explore alternative approaches that are effective, safe, and inexpensive in promotion of HPSC engraftment.

Photobiomodulation therapy (PBMT) applies a red-beam or near-infrared light with a wavelength ranging from 600 to 1100 nm and output power of 1–500 mW. The light can be a continuous wave or pulsed ones with relatively low energy density (0.04–50 J/cm^2^) and generates no significant heat, also called “cold laser therapy”. It has been clinically applied in wound healing^6,7^, tissue repair, pain relief^8^, and inflammation control^9^ for more than two decades with a long record of safety. Previous studies have shown PBMT is effective to promote proliferation of peripheral blood lymphocytes^10^ and bone morrow-derived mesenchymal stem cells^11^ and enhance the potential of long-term cryopreserved peripheral blood hematopoietic progenitor cells to grow in vitro^12^. Our recent studies have shown that PBMT promotes megakaryocyte differentiation from HSCs under the stress of thrombocytopenia in both in vivo and in vitro^13,14^. PBMT also extended a shelf-life of stored human platelets in preclinical study with improved functionality^15^. Given the well-documented effects of PBMT on the survival and differentiation of HSCs and other blood cells, we hypothesized that PBMT might have a beneficial effect on engraftment of HPSCs derived from hUCB.

We show here that a single PBMT of HSPCs before infusion significantly enhances engraftment of HPSCs in immunodeficient mice. Reactive oxygen species (ROS)/Src signaling pathway might be involved in this bio-modulation effect. These findings support PBMT to be potentially a safe, convenient, and cost-effective modality to facilitate a rapid hematological recovery in patients after UCB transplantation.

## Results

### PBMT promotes rapid engraftment of hUCB HPSCs in immunodeficient mice

**Figure 1:**
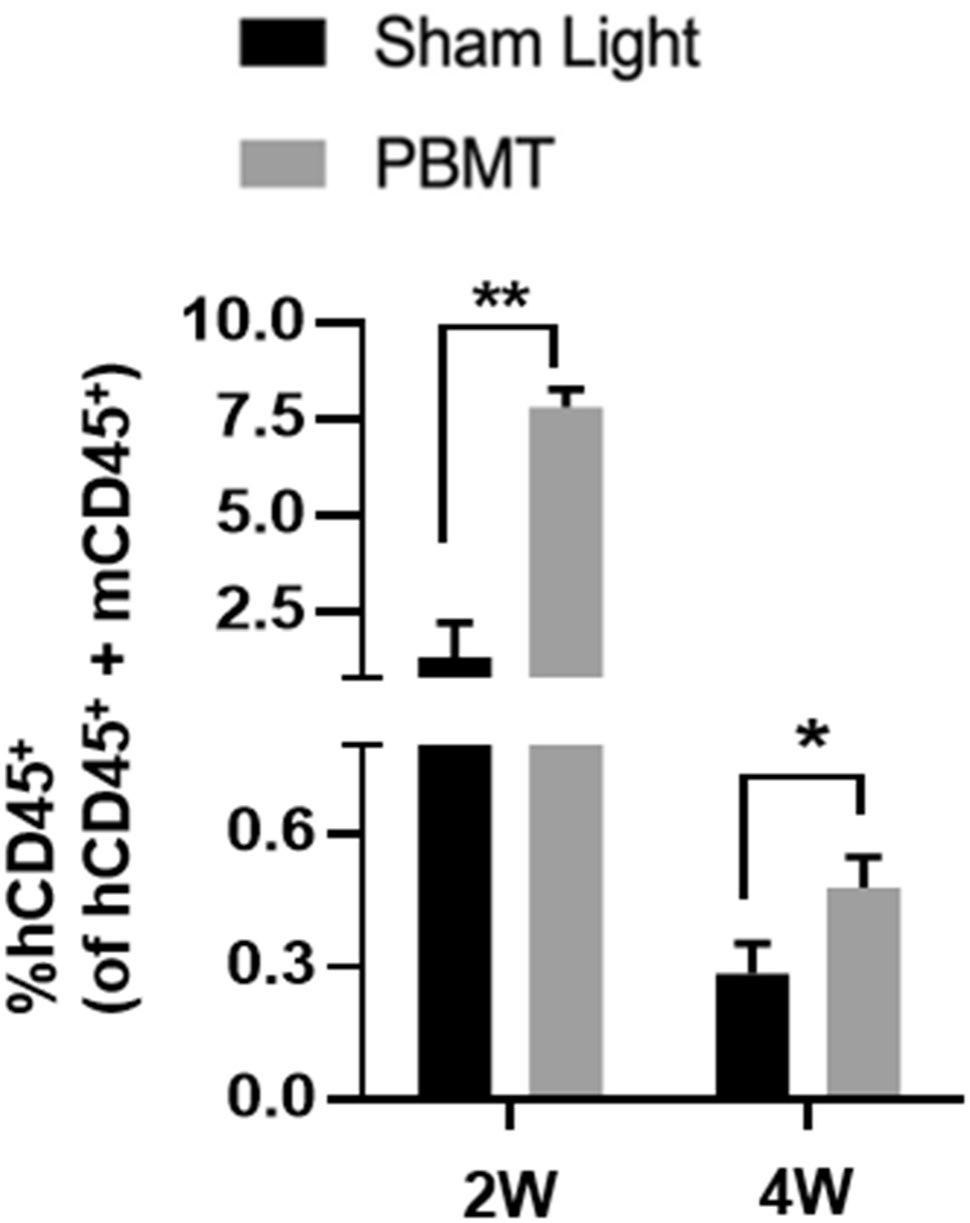
PBMT promotes engraftment of hUCB HPSCs in immunodeficient mice. Human hUCB CD34^+^ cells were irradiated with a LED array at 2 J/cm^2^, 830 nm and then transplanted into NSG mice. The percentages of human CD45^+^ cells relative to a total of CD45^+^ cells were assessed in peripheral blood at 2 and 4 weeks (W) post-transplantation. n = 5 in each group. *p < 0.05 and **p < 0.01 compared in the presence or absence of PBMT.

To investigate effects of PBMT on engraftment of HPSCs in immunodeficient mice, hUCB CD34^+^ HSPCs were treated with either sham or a single 830 nm light at 2 J/cm^2^ prior to infusion into immunodeficient NOD/SCID-IL2Rγ^−/−^ (NSG) mice. The 830 nm light was transmitted to CD34^+^ cells cultured in medium by an array of light-emitting diodes (LED). The immunodeficient mice were conditioned with a whole body γ-irradiation four hours prior. Peripheral blood cells were taken from the chimerical mice at indicated times and enumerated for human and mouse CD45^+^ cells by flow cytometry. As shown in Fig. 1, the mean percentages of human CD45^+^ blood cells were significantly higher in NSG mice receiving PBMT-treated CD34^+^ cells than those receiving sham-treated CD34^+^ cells when assayed at two and four weeks after infusion. Especially, at two weeks after infusion, the engraftment was threefold higher in PBMT-treated cells than controls. However, human blood cells declined in number over time and were no longer detectable after 6 weeks due to immune rejection of human cells by the mouse immune system (data not shown). These data suggest that a single PBMT of HPSCs just before infusion could significantly enhance the early engraftment of HSPCs.

### PBMT promotes ROS generation and Src tyrosine kinase activation in hUCB HSPCs

**Figure 2:**
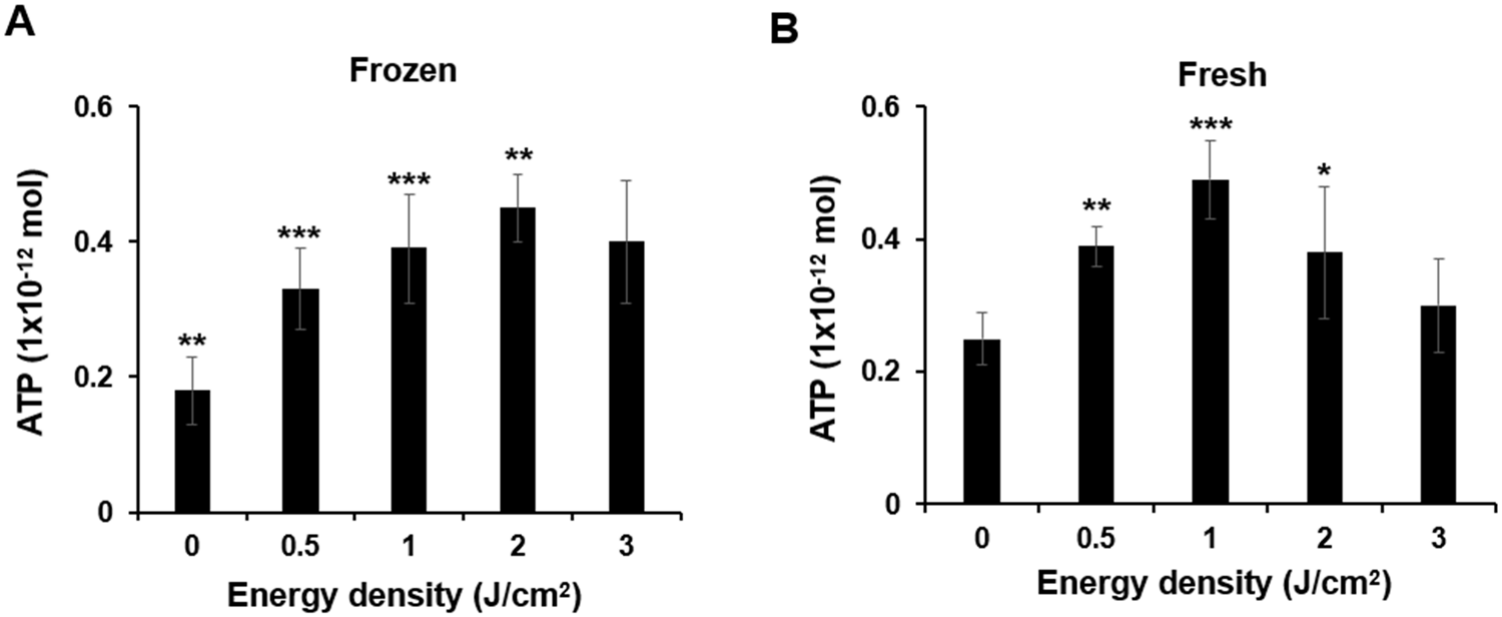
PBMT augments ATP production in frozen and fresh hUCB HPSCs. Frozen (**A**) and freshly isolated (**B**) hUCB CD34 + cells were treated with 830 nm at 0, 0.5, 1, 2, or 3 J/cm^2^ for 30 min, after which the cells were subject to ATP measurement. Data represent mean ± SEM. n = 6. *p < 0.05, **p < 0.01, and ***p < 0.001 compared in the presence or absence of PBMT.

**Figure 3:**
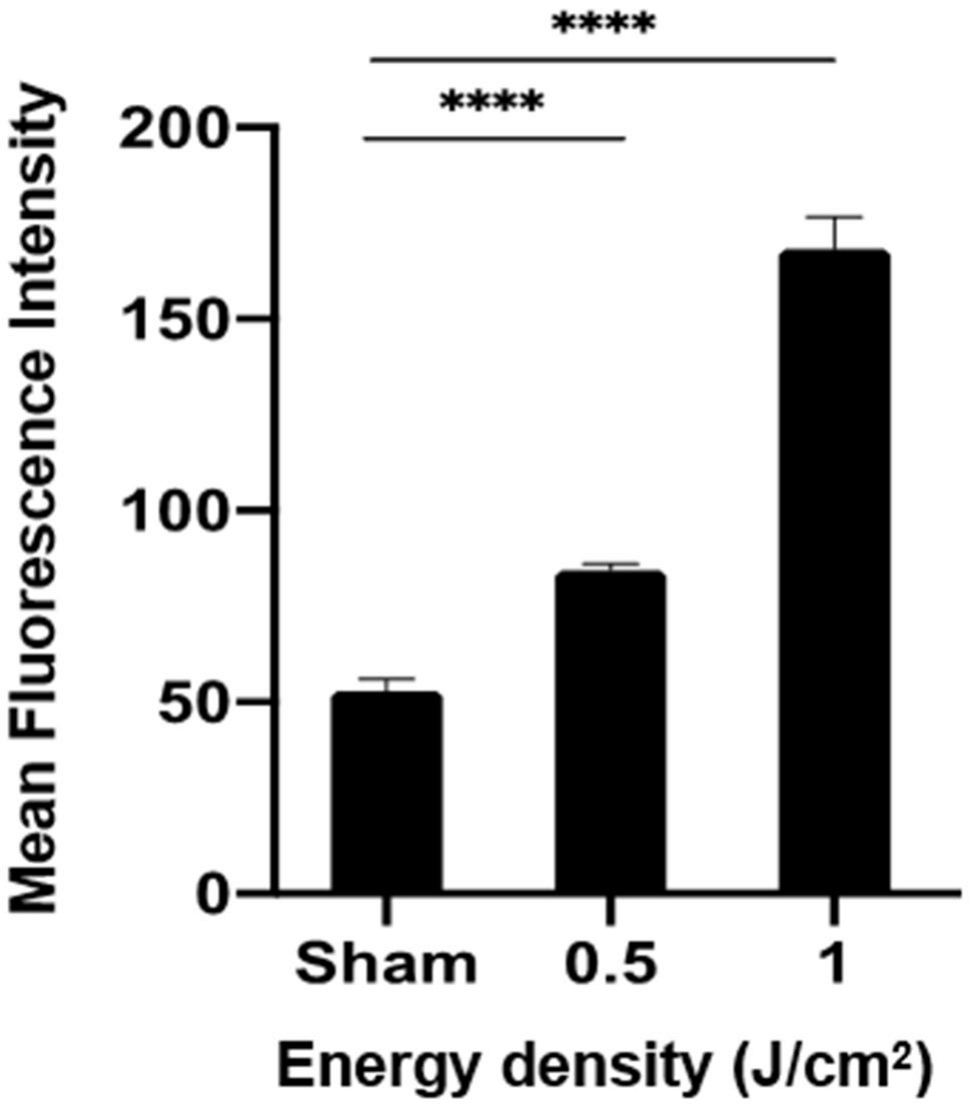
PBMT enhances ROS generation in hUCB HPSCs. CD34^+^ cells were freshly isolated from hUCB and irradiated with sham light, 0.5 J/cm^2^ or 1 J/cm^2^ 830 nm LED. Five minutes later, the cells were incubated with 10 µM DCFH-DA at 37 °C for 20 min. The mean fluorescence intensity of DCF was measured by flow cytometry. All data are presented as mean ± SEM. n = 6 and ****p < 0.0001 compared in the presence or absence of PBMT or between indicated groups.

To understand the beneficial effects of PBMT on HSPC engraftment at molecular levels, we isolated CD34^+^ cells from hUCB as they were readily available to us in a sufficient number for Western blotting analysis. To determine any difference between frozen and fresh CD34 + cells in response to PBMT, we measured ATP production following varying fluences of the light. ATP production is commonly used as a reference to compare PBMT’s effects in different cells in light of well-documented effects of PBMT on the protection of mitochondrial functions or mitochondrial membrane potential^14,16,17^. It was found that ATP production peaked at 1 J/cm^2^ in freshly isolated CD34^+^ cells, yet at 2 J/cm^2^ in frozen CD34^+^ cells (Fig. 2). Because of a bi-phase effect of PBMT, we subsequently chosen 1 J/cm^2^ for our in vitro mechanism study. We first measured intracellular ROS levels after the 830 nm LED irradiation. As shown in Fig. 3, PBMT increased the mean fluorescence intensity of ROS-sensitive DCFH-DA agent in CD34^+^ cells freshly purified from hUCB samples in a dose-dependent manner, suggesting that PBMT promotes the generation of intracellular ROS in hUCB HSPCs.

**Figure 4:**
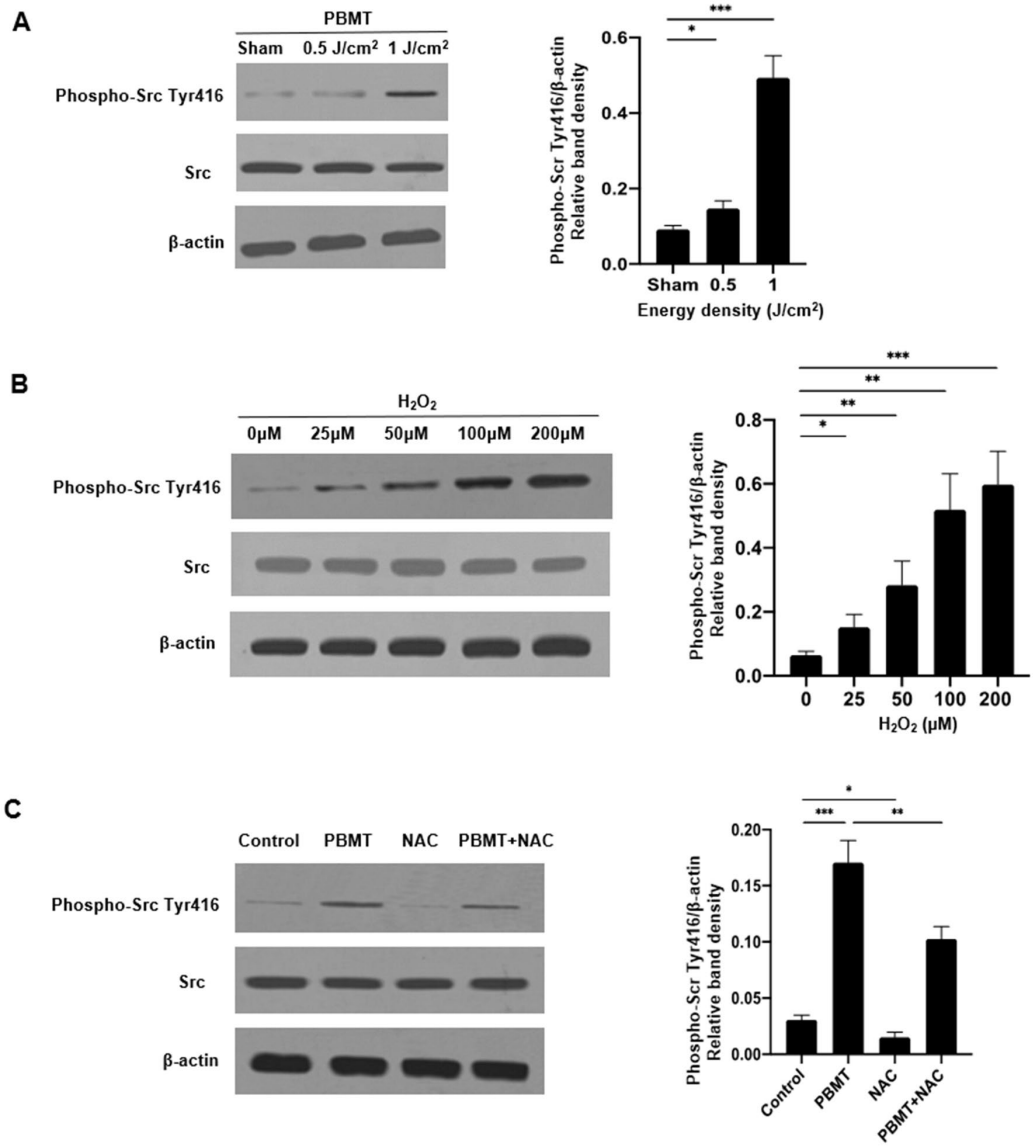
PBMT promotes Src tyrosine kinase activation in hUCB HPSCs via ROS. (**A**) CD34^+^ cells were irradiated with sham light or 0.5 J/cm^2^, 1 J/cm^2^ of 830 nm light. Five minutes later, Western blotting was performed with specific antibodies against phospho-Src Tyr416 , Src, or loading control β-actin. (**B)** CD34^+^ cells were treated by indicated concentrations of H_2_O_2_ for 30 min, and then subjected to Western blotting as (**A**). (**C**)CD34^+^ cells were treated with 1 mM of NAC for 30 min, and then irradiated with 1 J/cm^2^, followed by Western blotting as (**A**). Representative Western blots of three independent experiments are shown in the left and relative band densities (phospho-Src Tyr416/β-actin) were analyzed and were presented as mean ± SEM in the right. n = 3, *p < 0.05, **p < 0.01, and ***p < 0.001 compared in the presence or absence of PBMT or between indicated groups.

The effect of PBMT on Src tyrosine kinase (called Src hereafter) activation was next investigated. Src activity is regulated by tyrosine phosphorylation at two sites with opposing effects- tyrosine 527 (Tyr527) and tyrosine 416 (Tyr416). Phosphorylation of Tyr527 in the carboxy-terminal tail of Src by Csk inhibits the enzyme activity, whereas Tyr416 of Src can be phosphorylated during the activation of Src kinase, resulting in upregulation of Src kinase activity^18^. Tyr416 of Src phosphorylation was markedly enhanced by 830 nm LED irradiation in a dose-dependent manner after the treatment of 0.5 J/cm^2^ and 1 J/cm^2^ PBMT, as shown by Western blotting (left panel) and the corresponding density analysis of Src Tyr416 phosphorylation relative to control protein β-actin (right panel) (Fig. 4A). The results corroborate the ability of PBMT to enhance Src activation in hUCB HSPCs.

We next asked a role for ROS in PBMT-mediated Src activation. Freshly isolated CD34^+^ cells were treated by different concentrations of hydrogen peroxide (H_2_O_2_), followed 5 min later with measurement of Src Tyr416 phosphorylation. Tyr416 phosphorylation was significantly increased by H_2_O_2_ in a dose-dependent manner within a range from 25 µM to 200 µM (Fig. 4B). Moreover, when the cells were treated with 1 J/cm^2^ PBMT in the presence of 1 mM N-acetyl cysteine (NAC), an antioxidant, Tyr416 phosphorylation diminished significantly when compared to controls (Fig. 4C). The findings suggest that ROS/Src signaling pathway might play a role in promotion of engraftment of pre-treated hUCB HSPCs.

## Discussion

A delayed hematopoietic recovery is associated with a high risk of morbidity and mortality and how to achieve efficient engraftment of HPSCs remains a significant challenge for UCB transplantation. Here we demonstrate that a brief illumination of HPSCs with 830 nm LED before infusion greatly improves HPSC engraftment in humanized immunodeficient mice. In contrast to a laser beam of point-focus, the handheld LED array as a source of PBMT is safer and more cost-effective^19^. It can illuminate a relatively larger area uniformly and thus an entire hUCB unit or several units at same time in clinical practice. The novel approach can potentially result in a better hematopoietic reconstitution, compensate for a limited number of a single hUCB unit, and reduce the need for double-unit UCB transplantation. In comparison with single unit of hUCB infusion, double-unit transplantation is more commonly associated with complication after infusion and tedious donor-matching prior to infusion^3^.

PBMT can be a physiological stimulant for the proliferation and differentiation of stem cells at a wide range of light parameters^20^. For instance, Fekrazad et al. describe that fluences ranging from 0.7 to 4 J/cm^2^ and wavelengths of 600–700 nm are all appropriate to stimulate mesenchymal stem cells (MSCs)^21^. Nasciment et al. observed recently that PBMT conducted with a semiconductor laser at wavelength of 685 nm, power density (irradiance) of 2.6 mW/cm^2^, and energy density of 1 J/cm^2^ significantly enhanced the potential of long-term cryopreserved peripheral blood progenitor cells for in vitro growth^12^. Our previous study applied 810 nm diode laser as PBMT source and unraveled that PBMT at 3 J/cm^2^ could facilitate ATP generation of frozen human CD34^+^ cells and their differentiation into megakaryocytes and platelets^13^. When four wavelengths (420, 540, 660, and 810 nm) at an energy density of 3 J/cm^2^ were compared for their ability to promote the differentiation and proliferation of human adult stem cells, 420 and 540 nm laser promoted osteoblast differentiation and intracellular calcium influx better than 660 and 810 nm^22^. In the current study, PBMT at 2 J/cm^2^, 830 nm LED significantly promotes frozen hHSPC engraftment. Yet, freshly isolated CD34^+^ cells appear to be more sensitive to the light as compared to frozen cells, with an optimal response at 1 J/cm^2^. Perhaps, frozen cells suffer from more stress, a significant number of them may be dying via either necrosis or apoptosis, and the cells could be thus less sensitive to the light. The effect of this low energy density is consistent with aforementioned MSC and HSC studies^12,13,21^. It has been suggested that a high power density pairing with a low energy density yields better results of stem cell proliferation or differentiation, and the wavelength seems to have insignificant influences on the proliferation of stem cells^20^. Hence, a low radiation exposure of PBMT may be necessary to obtain an optimal stimulation of HSCs, and a moderate irradiance of an approximate 20 mW/cm^2^ may be more beneficial to HSCs, although further study is needed to confirm it.

Currently, the exact mechanism underlying the biomodulation effect of PBMT is not well known. It has been shown that ROS formation can be modestly increased by PBMT, which in turn activates some transcription factors as a result of PBMT-mediated improvement of mitochondrial electron transport activity^23,24^. A recent study showed that intracellular ROS expression in HSCs was correlated positively with neutrophil engraftment in patients after autologous HSC transplantation^25^. Apart from ROS-mediated signaling pathway, PBMT may also influence DNA methylation and orchestrate gene expression during stem cell differentiation^26^. In this study, we show that a single PBMT significantly increases intracellular ROS formation, similar to the modulating effects of PBMT on other cells^20^. The PBMT-mediated increase of intracellular ROS may be accounted, at least in part, for the more efficient HSPC engraftment.

Recent studies have demonstrated that Src-family tyrosine kinases could be activated by cellular oxidative events^27,28^. These kinases are pivotal to activate various downstream kinases such as the mitogen-activated protein kinase (MAPK) family, Akt, protein kinase C, and the epidermal growth factor receptor kinase^29^. Src protein is a key member of the Src family of protein tyrosine kinases and plays a crucial role in cell morphology, motility, proliferation, and survival^30^. Zhang et al. found that PBMT induced ROS-mediated Src activation in HeLa cells^31^. In agreement with this, we showed that PBMT activated Src also in association with increased ROS production in HSPCs. These results suggest a potential of Src activation in PBMT-facilitated engraftment of HSPCs. Further studies for the signaling pathways underlying this bio-modulation and clinical studies are warranted in the future.

## Methods

### Cells and mice

Frozen human UCB CD34^+^ cells were obtained from STEMCELL Technologies (USA) and used for the in vivo engraftment study. The thawed CD34^+^ cells were suspended in the serum-free expansion medium (SFEM) (STEMCELL Technologies, USA) at a concentration of 1 × 10^6^/mL and maintained at 37 °C in 5% CO_2_.

For in vitro studies, hUCB was obtained from full-term deliveries. Informed consent was obtained from all healthy adult donors. The study was carried out in accordance with the Declaration of Helsinki and approved by the Partners Human Research Ethics Committee of Massachusetts General Hospital (MGH). For isolation of CD34^+^ cells, mononuclear cells (MNCs) were first isolated by density gradient centrifugation using Ficolle-Hypaque Premium (GE healthcare, USA). Then, CD34^+^ cells were purified from MNCs using magnetic bead conjugated anti-CD34 antibody (Miltenyi Biotec, Germany) with the Magnetic Activated Cell Sorting method according to the manufacturer’s instructions (Miltenyi Biotec, Germany). The purified CD34^+^ cells were seeded in Iscove's Modified Dulbecco's Medium (IMDM) containing 1% fetal bovine serum (FBS; Thermo Fisher Scientific, USA) at a concentration of 1 × 10^5^/mL and maintained at 37 °C in 5% CO_2_.

NOD/SCID-IL2Rγ^−/−^ (NSG) mice at 4 weeks of age were obtained from the Jackson Laboratory (USA). All animal procedures were approved by the Institutional Animal Care and Use Committees (IACUC) of MGH in accordance with guidelines of the National Institutes of Health (NIH).

### Photobiomodulation therapy

PBMT was performed using a handheld Omnilux New-U device (Photo Therapeutics Inc., USA) that was composed of an array of LED with an area of 6 × 5 cm^2^ in size. The device delivered a near-infrared 830 nm light with a continuous wave and an irradiance of 33.2 mW/cm^2^. The cells to be illuminated were seeded in a 6-well culture plate (Thermo Fisher Scientific) and exposed to LED at an average irradiance of 20 mW/cm^2^. PBMT was tested at a fluence of 0.5 J/cm^2^, 1 J/cm^2^, 2 J/cm^2^, or 3 J/cm^2^, corresponding to an illumination of 25, 50, 100, and 150 s, respectively. The sham light was administered similarly with a small soft white LED light bulb (3 W, A15) (General Electric, USA).

### In vivo engraftment

CD34^+^ cells were treated with a single illumination of sham light or 830 nm, 2 J/cm^2^ transmitted by the LED array, then washed, and suspended in 0.9% normal saline. NSG mice were conditioned by whole body γ-irradiation (0.6 Gy/min, 2.5 Gy) 4 h before intravenous injection of 1 × 10^5^ CD34^+^ cells per mouse via the lateral tail vein. Peripheral blood was taken from the retro-orbital venous plexus into EDTA tubes under ether anesthesia every two weeks after the transplantation. For flow cytometry analysis, the collected peripheral blood samples were treated with red blood cell lysis buffer and then incubated with anti-human CD45-FITC and anti-mouse CD45-APC antibodies (BioLegend, USA) for 20 min on ice. Human cell chimerism was examined by the number of human and mouse CD45^+^ cells on FACSAria (BD Biosciences, USA). The percentage of human CD45^+^ cells was calculated relatively to a total number of CD45^+^ cells.

### Luminescent assay of ATP levels

Frozen human CD34^+^ cells or CD34 ^+^cells freshly isolated from hUCB were seeded in 96-well plate at 5 × 10^4^ cells per well in 100 μl of culture medium, to which 100 μl of CellTiter-Glo reagents (Promega) were added 30 min after LLL or sham light treatment. The cells were incubated at 37 °C for another 10 min and then the luminescence of each sample was measured on a microplate reader (Molecular Devices) per manufacturer’s instruction. All samples were tested in triplicate.

### Effects of H_2_O_2_ and NAC on Src phosphorylation

For H_2_O_2_ treatment, purified CD34^+^ cells were incubated for 30 min at 37 °C with 5% CO_2_ in IMDM supplemented with 25, 50, 100, or 200 mM H_2_O_2_ (Sigma-Aldrich, USA) and 1% FBS, then washed, and diluted in phosphate-buffered saline (PBS). For NAC treatment, purified CD34^+^ cells were incubated in IMDM supplemented with 1 mM NAC (Sigma-Aldrich, USA) and 1% FBS for 30 min at 37 °C in 5% CO_2_, then washed, and maintained in the cell culture medium. For Src phosphorylation assay, the cells were lyzed in a buffer containing 50 mM Tris–HCL, 150 mM NaCl, 1% NP-40, 0.25% Na-desoxycholate, 5 mM EDTA, 1 mM NaF, 25 mM Na_3_VO_4_, 0.1 mM PMSF and 2 mg/mL Aprotinin (pH 7.5). Cellular debris was pelleted at 12,000*g* for 5 min at 4 °C. Equal amounts of proteins (30 µg/well) were separated by 10% SDS-PAGE and transferred onto PVDF membranes. The membranes were blocked with 5% fat-free milk and incubated with antibodies specific for Src (1:1000 dilutions; Cell Signaling, USA), phospho-Src Tyr416 (1:1000 dilutions; Cell Signaling, USA), or β-actin (1:5000 dilutions; Cell Signaling, USA) at 4 °C overnight. Specific staining was visualized with enhanced chemiluminescence detection reagents (Thermo Scientific, USA) following incubation with horseradish peroxidase-conjugated secondary antibody. Individual protein bands in the Western blots were quantified using Image Studio Lite version 5.2 software (LI-COR Biosciences, USA). β-actin was used as the loading control for normalization.

### Intracellular ROS measurement

Intracellular ROS were measured by a nonfluorescent probe 2′,7′-dichlorofluorescein diacetate (DCFH-DA), which can passively diffuse into cells where it is deacetylated by intracellular esterase to release nonfluorescent 2′,7′-dichlorofluorescein (DCFH). Oxidation of DCFH by ROS yields a highly fluorescent compound dichlorofluorescein (DCF) that is trapped inside the cells^32^. The resultant fluorescence intensity of DCF is proportional to the amount of intracellular ROS. In brief, cells were exposed to different energy densities of 830 nm LED irradiation followed by incubation with 10 µM DCFH-DA (Abcam, UK) at 37 °C for 20 min in the dark. The fluorescence intensity of DCF was measured by flow cytometry.

### Statistical analysis

Statistical significance was assessed by two-tailed student’s *t* test for two-group comparison using Graphpad Prism 8.0 (Graphpad Software). A value of *p* < 0.05 was considered statistical significance.

## Supplementary information

Supplementary Information 1.
