# Supplementary material for: 830 nm photobiomodulation therapy promotes engraftment of human umbilical cord blood-derived hematopoietic stem cells
Source: Sci Rep. 2020 Nov 12;10:19671. doi: 10.1038/s41598-020-76760-5 (PMC7661704; doi:10.1038/s41598-020-76760-5)
Supplement: Supplementary file 1 — Supplementary Information 1. [file 41598_2020_76760_MOESM1_ESM.docx]

**Title:**

830 nm photobiomodulation therapy promotes engraftment of human umbilical cord blood-derived hematopoietic stem and progenitor cells

**Authors and Affiliations:**

Jingke Yang, Li Wang, and Mei. X Wu*

Wellman Center for Photomedicine, Massachusetts General Hospital, Department of Dermatology, Harvard Medical School, Boston, MA 02114, USA

^*^Correspondence author. Email: mwu5@mgh.harvard.edu

**
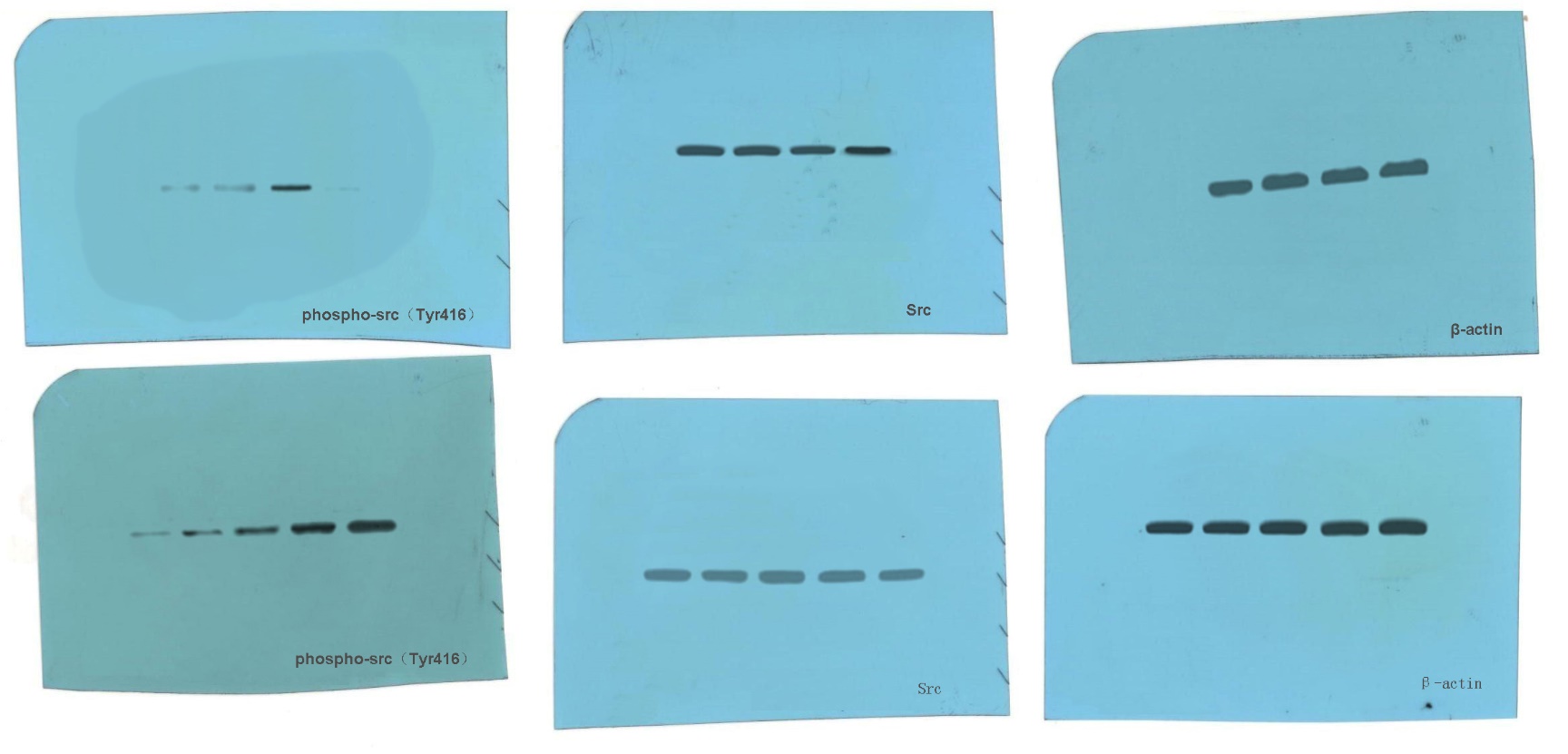
Figure 4A- Phospho-src-Tyr416**

**Figure 4A- Src**

**
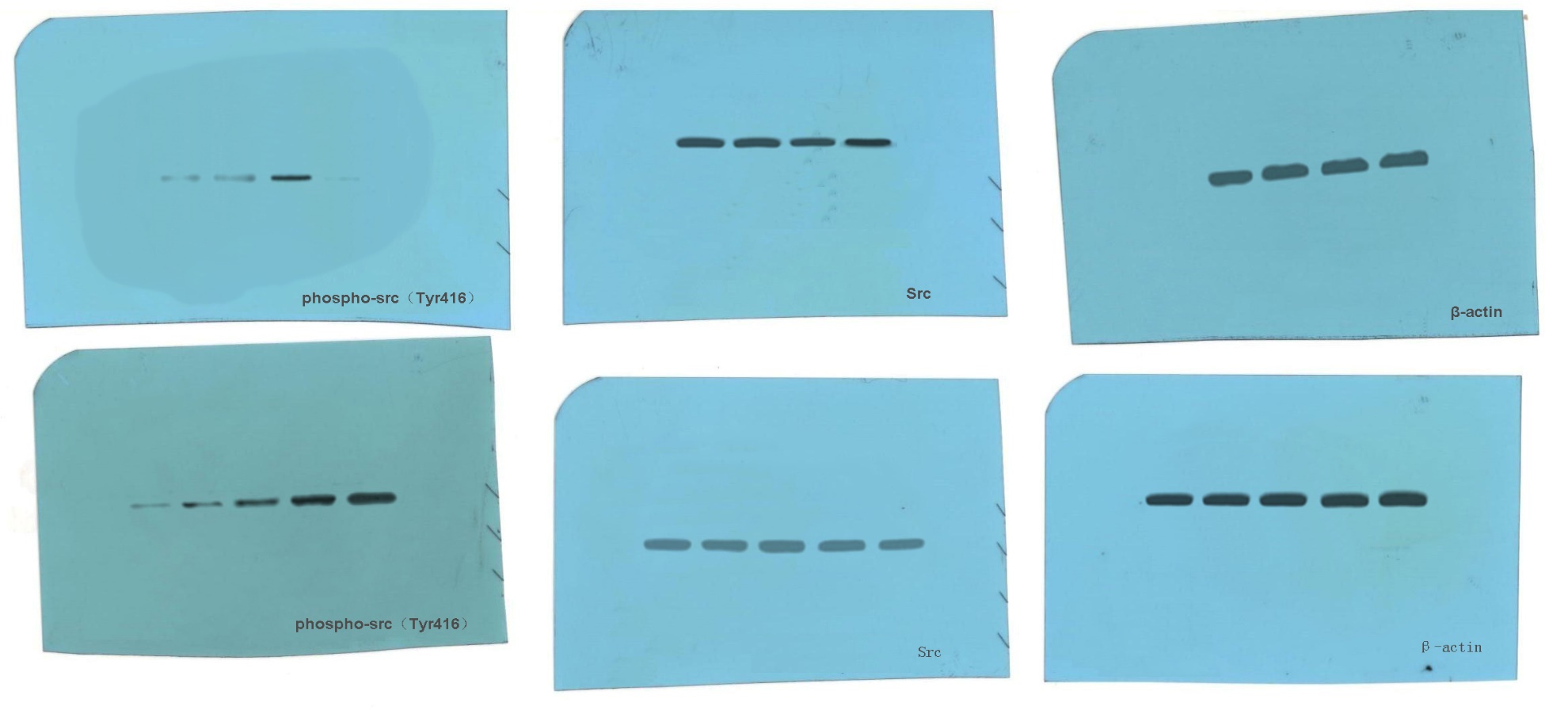
**

**Figure 4A- β-actin**

**
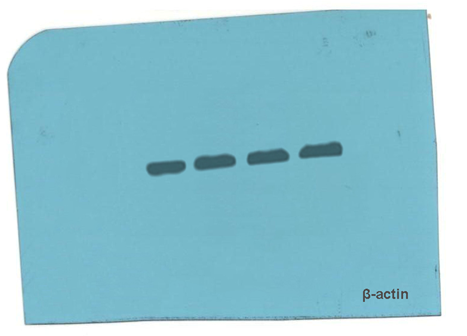
**

**Figure 4B- Phospho-src-Tyr416**

**
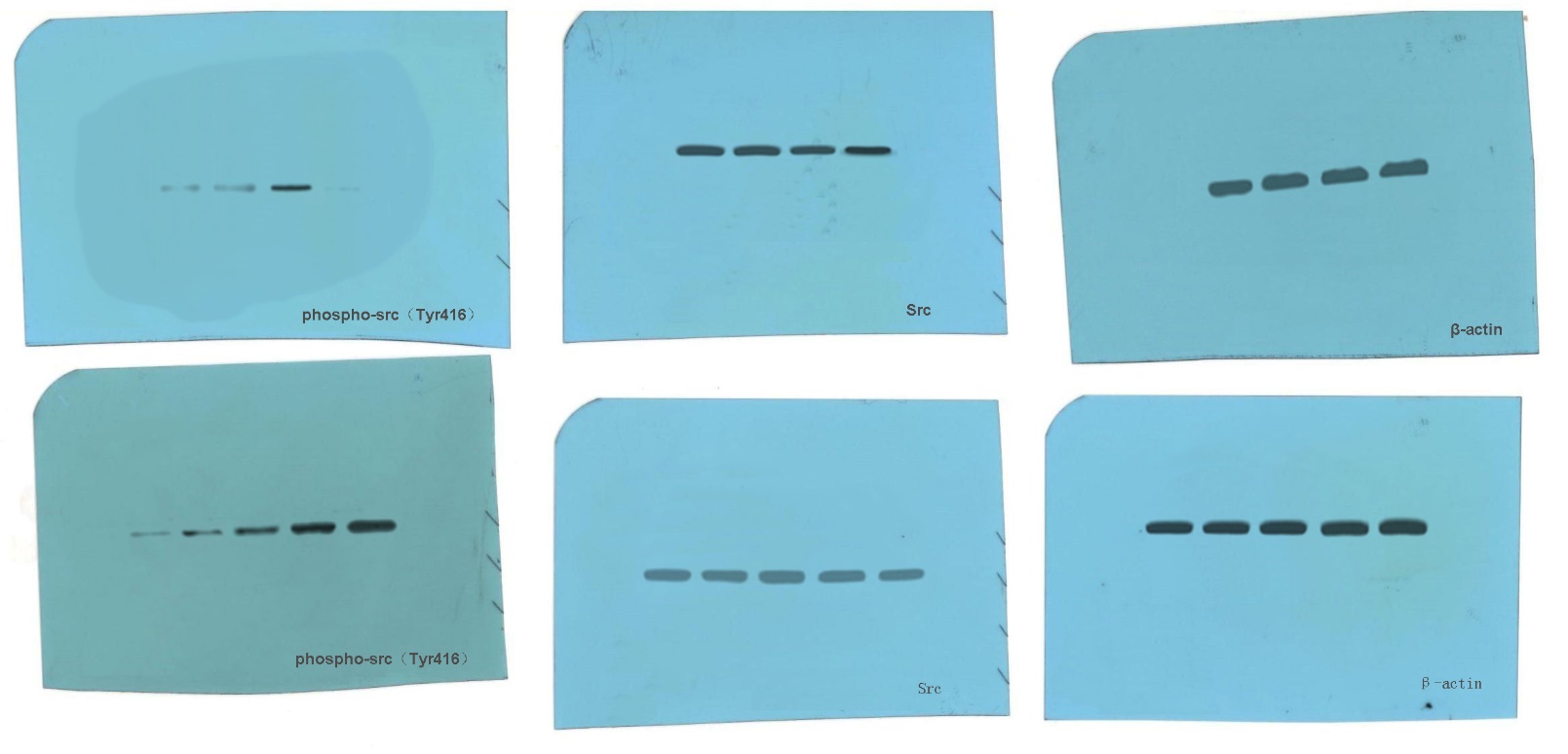
**

**Figure 4B- Src**

**
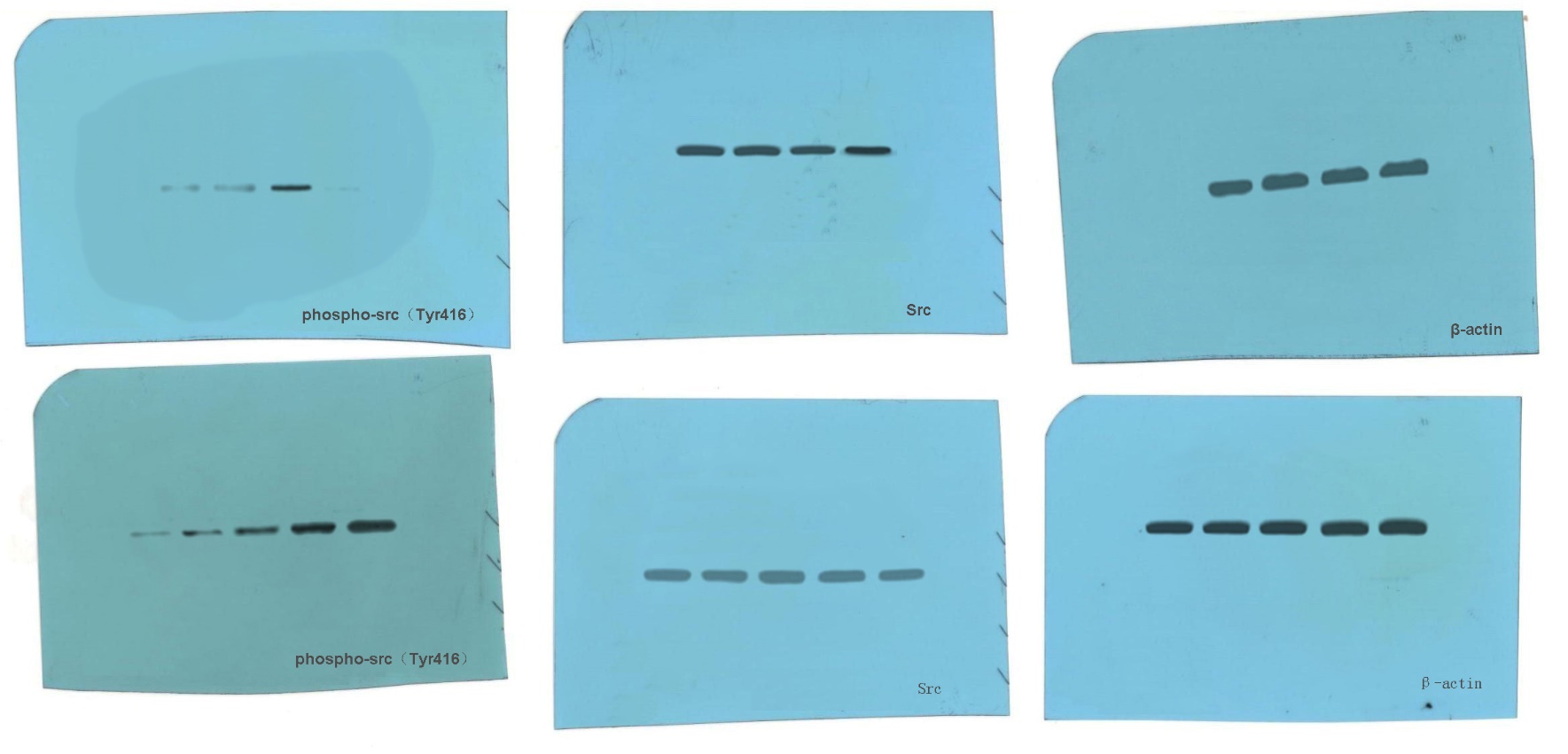
**

**Figure 4B- β-actin**

**
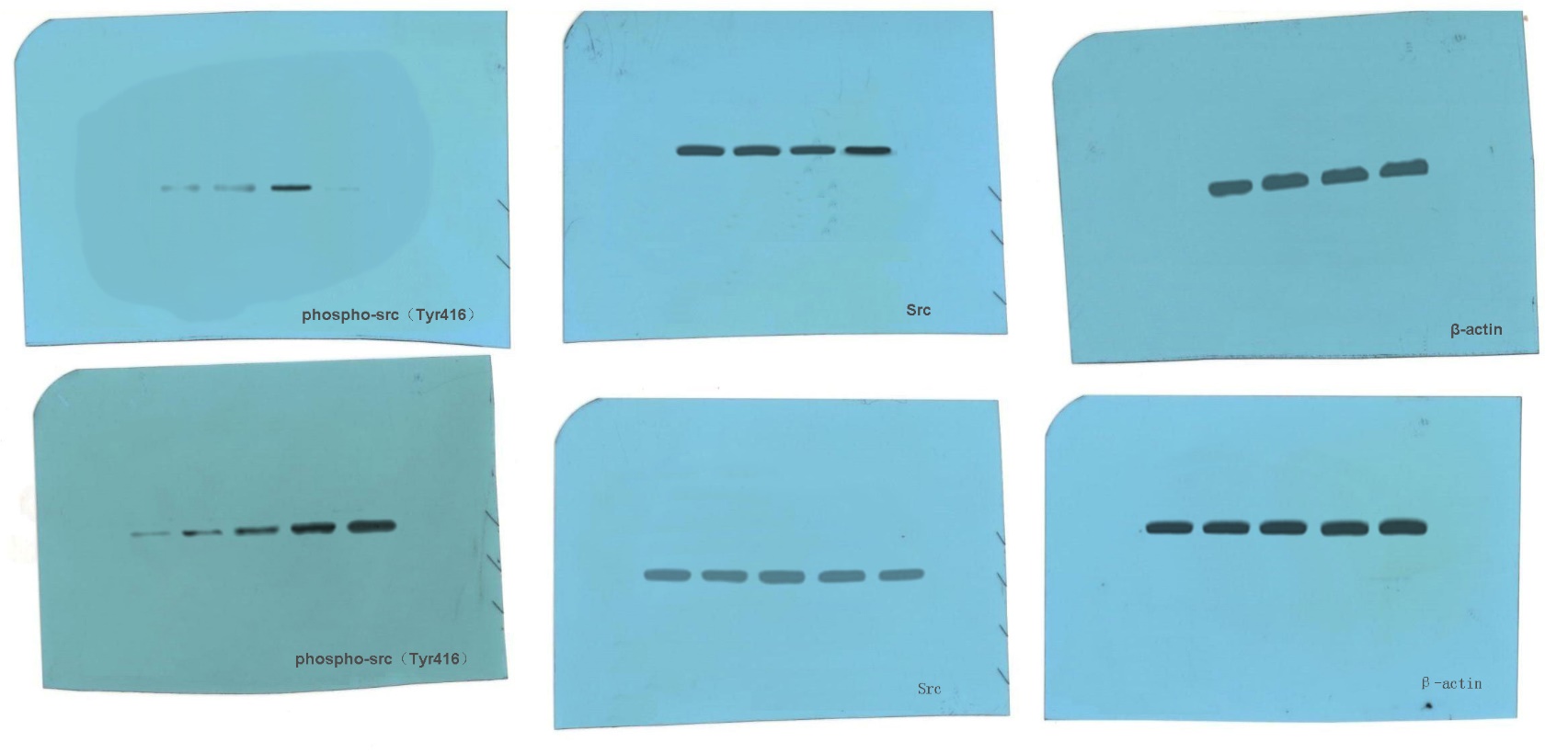
**

**Figure 4C- Phospho-src-Tyr416**

**
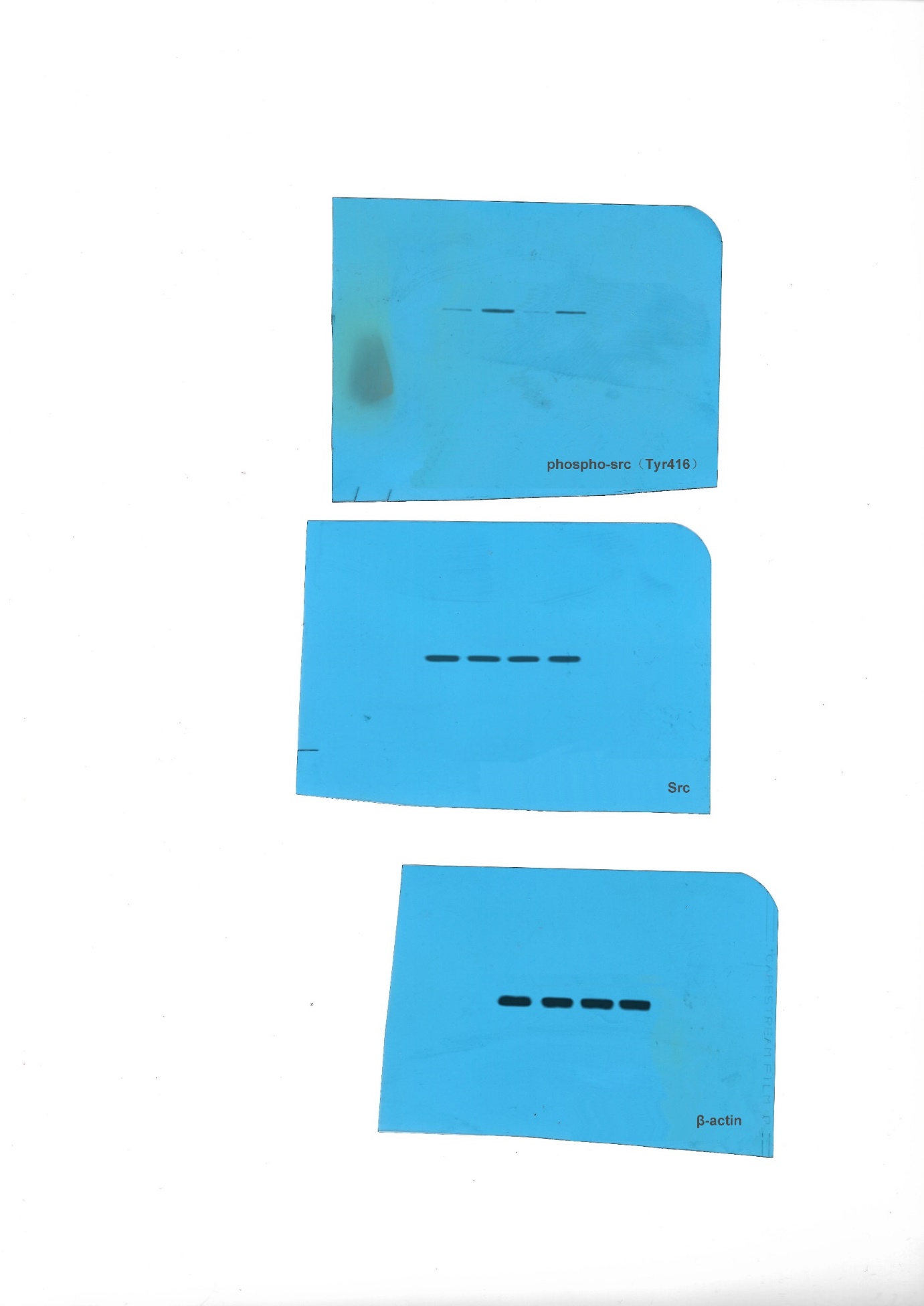
**

**Figure 4C- Src**

**
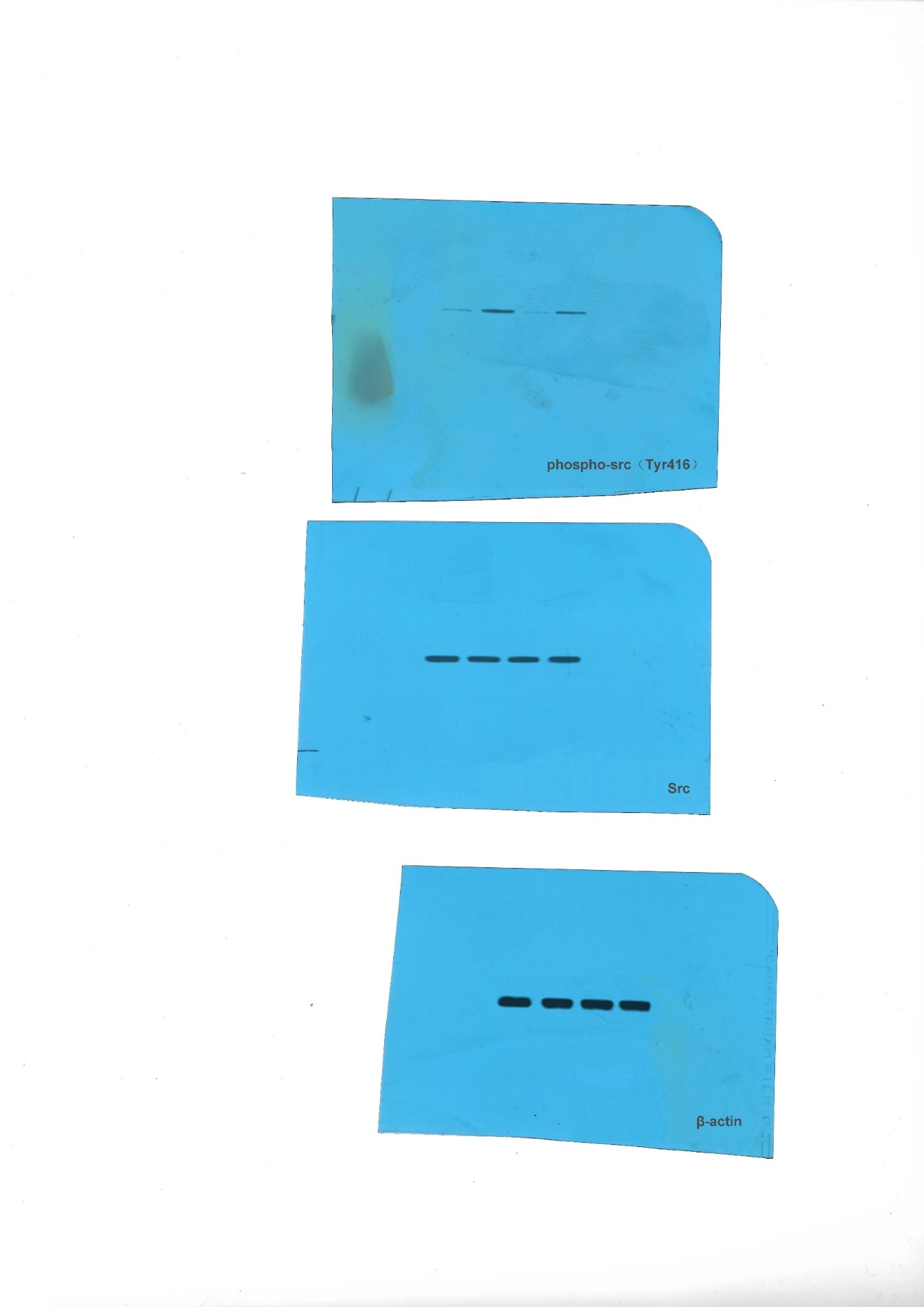
**

**Figure 4C- β-actin**

**
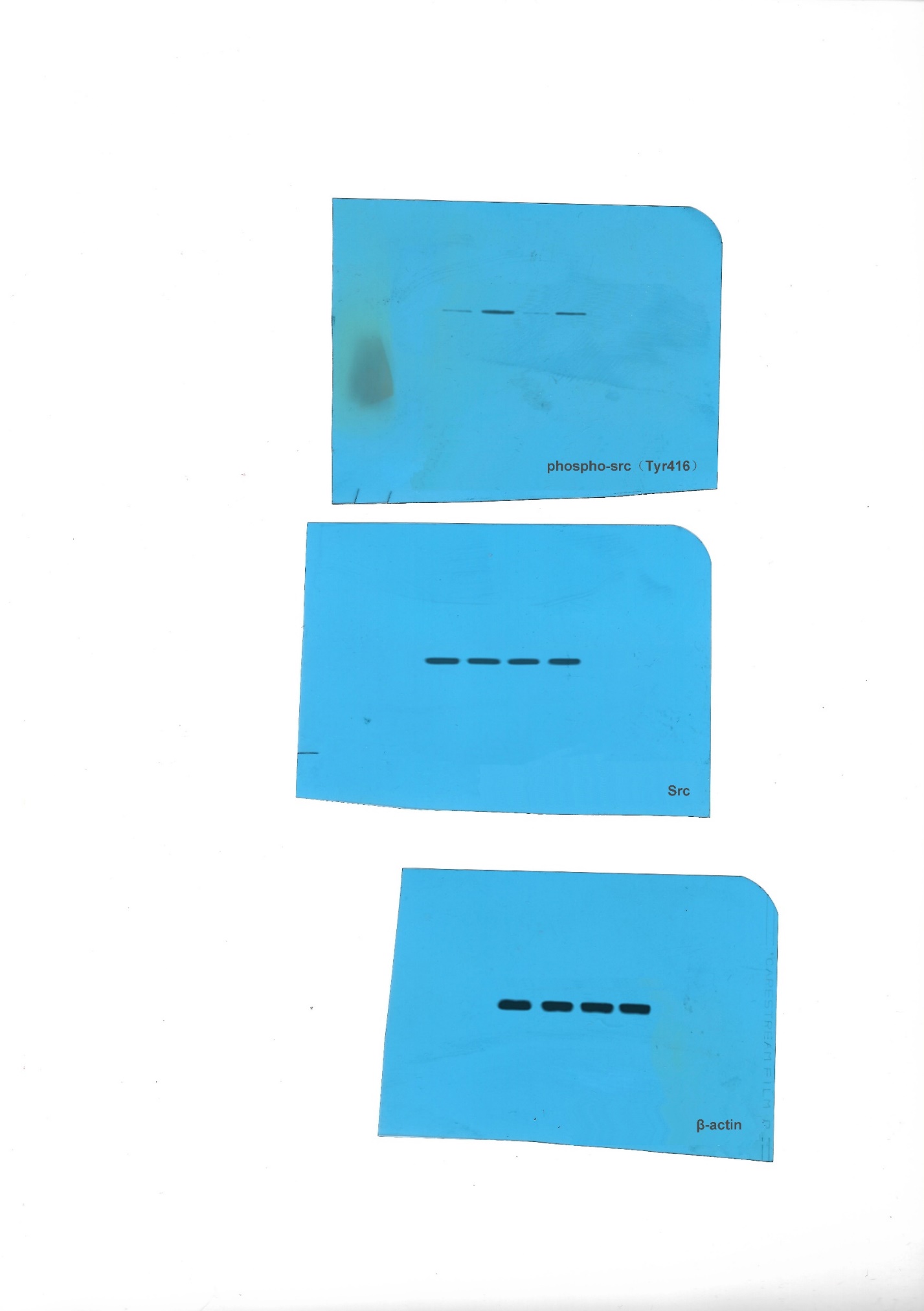
**
